# Transcriptomic Responses to Salinity Stress in the Pacific Oyster *Crassostrea gigas*

**DOI:** 10.1371/journal.pone.0046244

**Published:** 2012-09-27

**Authors:** Xuelin Zhao, Hong Yu, Lingfeng Kong, Qi Li

**Affiliations:** Fisheries College, Ocean University of China, Qingdao, Shandong, China; Biodiversity Insitute of Ontario - University of Guelph, Canada

## Abstract

**Background:**

Low salinity is one of the main factors limiting the distribution and survival of marine species. As a euryhaline species, the Pacific oyster *Crassostrea gigas* is considered to be tolerant to relative low salinity. The genes that regulate *C. gigas* responses to osmotic stress were monitored using the next-generation sequencing of whole transcriptome with samples taken from gills. By RNAseq technology, transcript catalogs of up- and down-regulated genes were generated from the oysters exposed to low and optimal salinity seawater.

**Methodology/Principal Findings:**

Through Illumina sequencing, we reported 1665 up-regulated transcripts and 1815 down-regulated transcripts. A total of 45771 protein-coding contigs were identified from two groups based on sequence similarities with known proteins. As determined by GO annotation and KEGG pathway mapping, functional annotation of the genes recovered diverse biological functions and processes. The genes that changed expression significantly were highly represented in cellular process and regulation of biological process, intracellular and cell, binding and protein binding according to GO annotation. The results highlighted genes related to osmoregulation, signaling and interactions of osmotic stress response, anti-apoptotic reactions as well as immune response, cell adhesion and communication, cytoskeleton and cell cycle.

**Conclusions/Significance:**

Through more than 1.5 million sequence reads and the expression data of the two libraries, the study provided some useful insights into signal transduction pathways in oysters and offered a number of candidate genes as potential markers of tolerance to hypoosmotic stress for oysters. In addition, the characterization of *C. gigas* transcriptome will not only provide a better understanding of the molecular mechanisms about the response to osmotic stress of the oysters, but also facilitate research into biological processes to find underlying physiological adaptations to hypoosmotic shock for marine invertebrates.

## Introduction

The marine environment is changing rapidly around the world due to global warming [1]. During the last 10–15 years, glaciers and ice caps have been rapidly disappearing and more frequent intense rainfall events have been happening [1]. Owing to the huge amount of freshwater inflowed, the seas and oceans are disproportionately affected. Salinity of the superficial water and the inshore water decreased acutely in rainy season, which can incur the increasing mortality outbreaks and distribution shifts of marine species [2]. Salinity is a limiting factor to the survival and distribution of many marine organisms, especially as it varies downward [3]. Most marine invertebrates, as osmoconformers, have blood osmolarities close to that of seawater, lacking the ability to regulate the osmotic pressure of the internal medium [4]. It is doubtful whether changes in amino acid concentration are rapid enough to prevent cellular swelling in animals exposed to seawater whose salinity dropped abruptly [5]. Most marine invertebrates such as molluscs [6]–[8] and echinoderms [9] were demonstrated to suffer large-scale mortality when the salinity dropped below 20‰. Therefore, to certain aquatic economic species such as blue mussel [10], salinity fluctuation owing to rainfall in summer brought huge economic losses to aquaculture.

The Pacific oyster *Crassostrea gigas* is a dominant species in many intertidal locations as well as an important aquacultured bivalve species. Summer mortality of the Pacific oyster attracted extensive attention [11], [12] around the world. Salinity fluctuate is a considered factor without doubt. *C. gigas* grows in optimal salinity ranged from 20‰ to 25‰, whereas they can occur at salinities below 10‰ and will survive salinities in excess of 35‰[13]. Given its euryhalinity, the Pacific oyster, as a marine mollusc, is a good model for studies of hypoosmotic stress. Considerable efforts have been invested so far in the mechanisms of salinity adaptations, and osmolytes had been proved to play a primary role in the osmotic activities of the Pacific oyster, such as the large amount of nitrogenous solutes, against the fluctuating extracellular osmolality [14]. Free amino acids had been identified to be important as intracellular osmolytes in *C. gigas* and contribute to the hypoosmotic adaption [15]. Moreover, molecular studies about the genes that are in relation to cellular osmo-regulatory mechanism of the oyster had been conducted [16], [17]. However, previous studies only covered several genes that regulate the individuals to adapt to the extracellular osmolality. The molecular mechanisms of osmo-adaptation remained unknown.

Recently, the next-generation sequencing technologies make large-scale sequencing possible by high-throughput and cost-efficiency [18]. The development of novel high-throughput DNA sequencing methods has provided a new means of both mapping and quantifying transcriptome. The method, known as RNAseq, has clear advantages over existing approaches with sequencing depth at least 5 orders of magnitude [19] and highly accurate for quantifying expression levels [20]. To date, transcriptomes have been sequenced for various marine bivalves, such as clam, yesso scallop, vent mussel and oyster [21]–[25]. The countable, almost digital, nature of RNAseq data makes them particularly attractive for the quantitative analysis of transcript expression levels, which can give reliable measurements of transcript levels in one or more conditions [18]. By now, the next-generation sequencing technologies have shown probabilities to expanding sequencing database of more and more species, and influenced the analysis of gene regulation highly.

In the present study, we examined the whole transcriptome responses to low salinity stress of the Pacific oyster for the first time using the Illumina's sequencing technology. Considering individual monitoring of the oyster responses to salinity stress, two libraries were established from the gills of oysters that exposed to optimal and low salinity seawater, respectively. The study aimed to compare the expression patterns of the two libraries to better understand the transcriptomic regulation in oyster to low salinity stress and identify genes involved in osmoregulation of the Pacific oyster. The results of this study are an important resource for future researches on mechanism of tolerance to hypoosmotic stress for marine invertebrates.

## Results

### Sequencing and assembly

**Figure 1:**
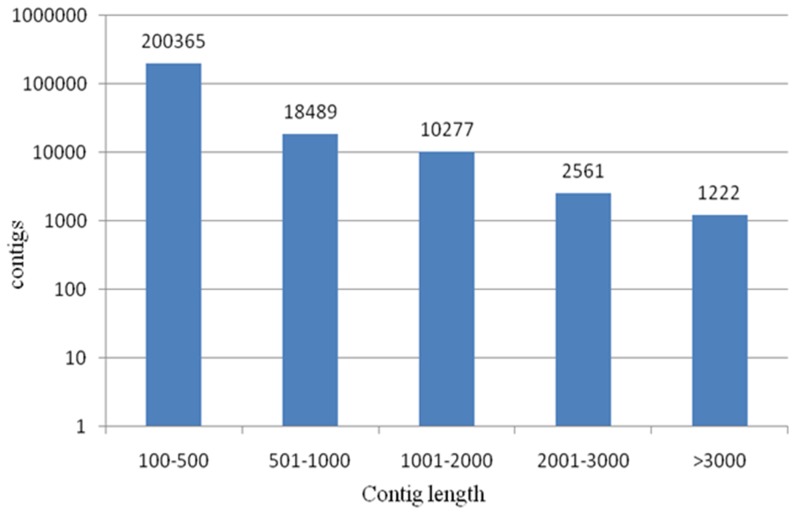
Distribution of number of read per contig in both libraries. The number of contigs presenting the indicated amount of reads is plotted as histogram.

To have access to the transcriptome of the oyster and obtain the quantitative and qualitative gene expression database for hypoosmotic stress, two libraries were established from the control group (PC library) and the treated group (PT library). Characteristics of the two libraries are summarized in **Table 1**. A total of 15,354,006 raw nucleotide reads, with an average length of 93 bp, were produced from the control group, while 13,719,859 raw nucleotide reads, with an average length of 92 bp were produced from the treated group. After preprocessing to remove low quality reads, 99.24% clean reads of PC and 98.93% clean reads of PT were left and thus submitted for assembly. Through assembly and elimination of redundancy, 300,382 contigs were generated from both libraries (GEO accession numbers GSM932404 and GSM932405) with average length of 322 bp, and 32,549 contigs were longer than 500 bp. The distribution of contig length is shown in **Figure 1**, varying from 100 bp to more than 3000 bp. We conducted genes at the different expressions between PC library and PT library. In accordance with statistical convention, all P-values were adjusted with a false-discovery rate (FDR) correction for multiple testing by the Benjamini-Hochberg method [26]. According to P-value (0.001), comparison of the reads occurrences between the two libraries revealed that 3480 unique tags were differentially represented between the libraries (the absolute value of fold change >2, composed of 1815 contigs more represented in PC library and 1665 contigs more represented in PT library).

**Table 1:** Summary statistics of the transcriptome sequencing and assembly for *C.gigas* from control group (PC) and treated group (PT).

	PC	PT
Raw sequencing reads	15,354,006	13,719,859
Unique contigs	224,215	212,830
Contigs differentially represented (≥2 fold change)	4,118	5,619

### Functional annotation

The contigs were annotated taking into consideration the identity of the translation frame, and 281,800 contigs were predicted to mapping to protein-coding sequences by GetORF of EMBOSS firstly. Then, the putative protein-coding sequences used Blastp searching on the Swiss-Prot database, the NCBI non-redundant (Nr) protein database and the genome of *Aplysia californica* (GenBank Accession: AASC00000000) (E-value<1e-5). We acquired 45,771 putative proteins that can be annotated matching biological function. In addition, sequences without annotations may represent poorly conserved region (e.g., un-translated regions) in *C. gigas*.

**Figure 2:**
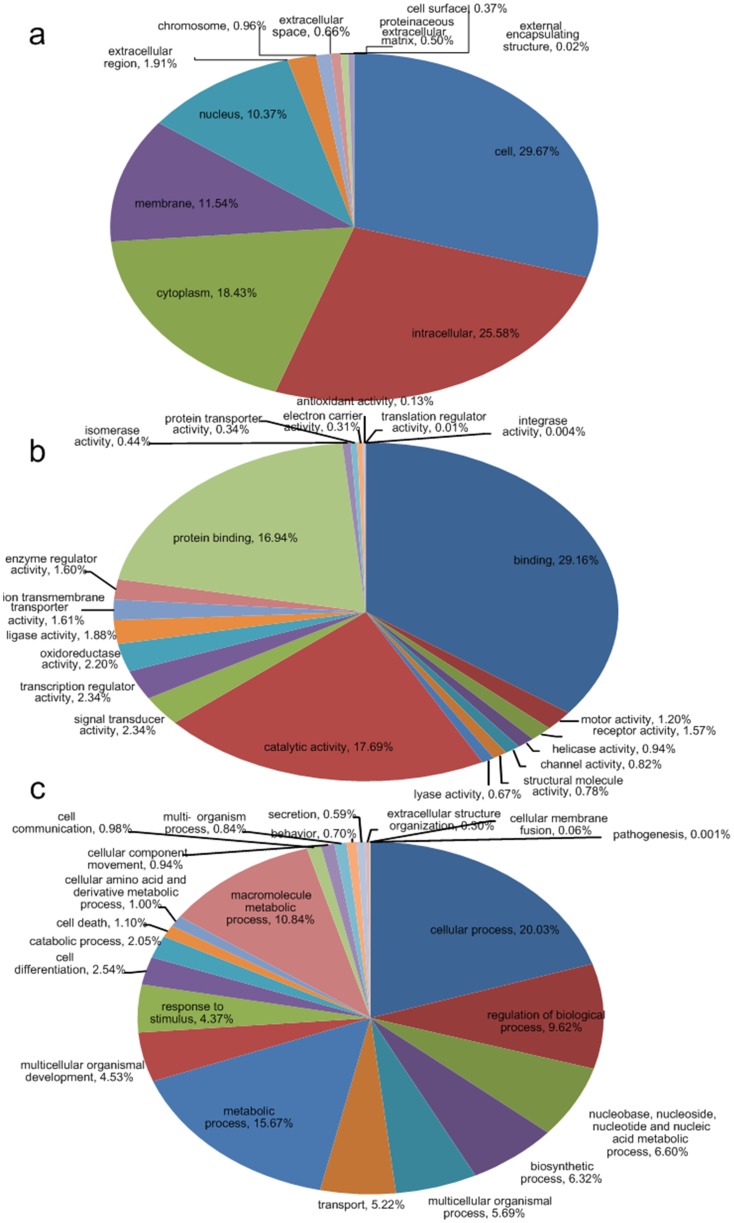
Classification of the annotated amino-acid sequences. Amino-acid sequences were grouped into different functional sub-categories: a cellular component b molecular function c biological process.

**Figure 3:**
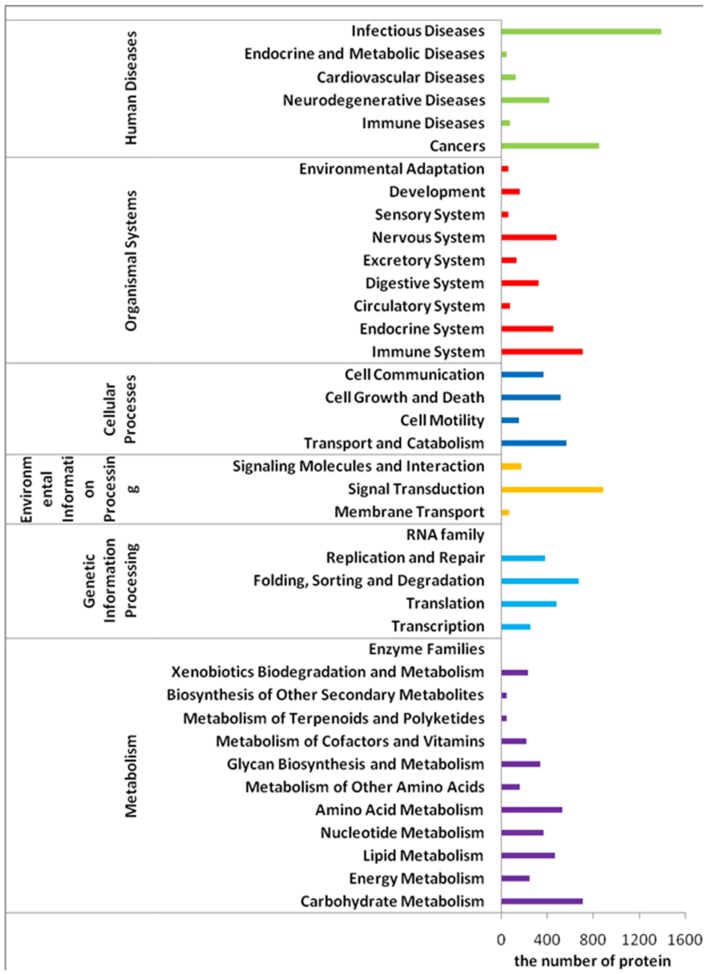
Distributions of the KEGG pathways. Putative proteins were mapped the reference canonical pathways in the KEGG database. The bar chart shows the number of sequences among different pathway categories: Out of 45,771 putative proteins, 5135 sequences were assigned to the KEGG pathways among six categories.

Secondly, Gene Ontology (GO) analysis was carried out, which conducted on the putative proteins blasting the Swiss-Prot and its supplement TrEMBL database. Of 45,771 putative proteins, 16,291 unique sequences were assigned with one or more GO terms, and 113,677 GO assignments were finally obtained. All GO assignments fell into broad categories for all three major GO functional domains (Biological Processes, Cellular Components and Molecular Function) as presented in **Figure 2**. Besides GO terms, KEGG pathway mapping based on KO terms for assignments was also carried out, and 5135 putative proteins were assigned to KO terms and mapped the reference canonical pathways in the KEGG database, which were shown in **Figure 3**. In addition, the statistical results indicated that there were significant changes (FDR<0.001) in the contigs of different expression out of all the contigs in 4 GO terms (“extracellular space”, “extracellular”, “structural molecule activity” and “cell motility”) and 2 pathways (“immune disease” and “energy metabolism”). The 3480 unique transcripts differentially represented between libraries (>2-fold change) were annotated into functional groups based on GO terms and KO terms respectively. Among up- and down-regulated transcripts in the PT library, for molecular functions, transcripts involved in binding (GO: 0005488) and protein binding (GO: 0005515) were highly represented; for cellular component, transcripts involved in intracellular (GO: 0005622) and cell (GO: 0005623) were most represented categories. Whereas, regarding biological processes, to up-regulated tanscritpts, cellular process (GO: 0009987) were the most represented GO term, followed by regulation of biological process (GO: 0050789) and cell communication (GO: 0007154), and to down-regulated transcripts in the PT library, the most represented category was cellular process.

### Biological functions related to oyster response to hypoosmotic stress

We focused on biological functions that may characterize the responses to hypoosmotic stress. Comparing their occurrences in PC *versus* PT library, a list of transcripts has been established that included different expressions to responses to salinity stress (**Table S1** and **Table S2**). **Osmoregulation** is implicated by the up-regulation of the potassium channel tetramerisation domain containing 1 (KCTD1), NFX1-type zinc finger-containing protein 1 and Cysteine-rich secretory protein Mr30, and the down-regulation of the ion and amino acid transporter genes, such as transient receptor potential cation channel subfamily M member 8 (LTrpC-8), neutral and basic amino acid transport protein rBAT and Na/Pi-cotransporter. In addition, signaling and interaction molecules such as cAMP-responsive element-binding protein-like 2, LRIG3 were evidenced as well as recognition molecules such as lectins. Besides, stress proteins such as heat shock proteins were found. **Immune response** is characterized by genes related to signaling pathways notably involved in innate immunity, which includes the up-regulated genes such as C-type lectin, thioester-containing protein and C1q domain containing proteins. In addition, the genes related to antimicrobial immunity are down-regulated including 2′–5′ oligoadenylate synthetase 1 and zinc proteinase. Apoptosis is noticed with not only regulating anti-apoptotic genes such as the up-regulation of Baculoviral IAP repeat-containing protein, but also apoptosis triggers such as the down-regulation of caspases, programmed cell death 1 ligand 1 (PD-L1). **Cell adhesion and communication** is mainly in down-regulated genes such as collagen and integrins that related to cell membrane molecules. **Cytoskeleton** is organized by the up-regulated expression of actin, tubulin and ankyrins. In addition, genes coding for receptors associated to cytoskeleton network are also up-regulated. **Cell cycle** is dominated by up-regulated genes related to promote cellular proliferation like protein fosB and MYC homolog. Besides, MEGF 10 is a negative regulator of cell cycle and down-regulated. Interestingly, **Ca^2+^-binding proteins** were highlighted, owing to their significantly down-regulated expression. Ca^2+^-binding proteins included protocadherin, calmodulin, copine family proteins and calcium-binding EGF domain protein that combine with Ca^2+^ for the control of signaling processes, defense responses and other biological processes.

## Discussion

In this study, we compared the Pacific oyster transcriptomes of two groups, one in optimal salinity seawater (25‰), and the other in low salinity seawater (8‰). Our data show significant changes of up- or down-regulated genes between two groups, which indicate the putative molecular mechanism of adaption to hypoosmotic shock in *C. gigas*. It is noteworthy that *C. gigas*, as a marine shelled mollusc, keeps up the osmotic disbalance with the environment by means of temporary inhibition of the water-salt exchange to seal mantle cavity [4]. We have avoided the effect of closed shell by chipping away a part of shell edge of each oyster.

Functional annotation of genes differentially represented between PT and PC libraries has revealed biological processes that may characterize the responses to salinity stress. From the data, we found enrichment for genes related to signal transduction, cell adhesion and communication, ion channels and so on, implicated in the regulation of hypoosmotic stress response. Moreover, cellular processes related to immune response, cell cycle and differentiation, cytoskeleton rearrangements, as well as anti-apoptotic processes, were all highly represented.

Similar to most marine molluscs [4], [27], *C. gigas*, regulates the intracellular concentration of solutes to adapt to the surrounding conditions [16], [28]. Ion channels are the direct regulators and discovered changed in our data, such as KCTD 1, Cysteine-rich secretory protein Mr30. KCTDs, the N-termini of KCTD proteins and some voltage-gated K^+^ (Kv) channels are homologous. With four Kv channel subunits aggregating to create a transmembrane ion conduction pathway, KCTDs had been anticipated to bind to and regulate Kv channels [29]. Cysteine-rich secretory protein Mr30 known as an ion channel blocker that targets the ion channels [30], which could inhibit inorganic ions effusing from cytoplasm to stop active transport of these solutes into the cytosol, where the solute concentration is too high. Our discoveries may indicate that the up-regulation of ion channels allows ions to move freely from cytoplasm to external environment and inhibits water influx to balance osmotic pressure on either side of the membrane. LTrpC-8 was down-regulated in *C. gigas*, which mediates the permeation for cations such as sodium, potassium, calcium [31]. Another down-regulated gene in this context, Na/Pi-cotransporter, contributes to phosphate homeostasis that is dependent on presence of sodium [32]. While these expressions of down-regulated genes stop active transport of these solutes from the cytoplasm. These findings verify the results of previous studies and demonstrate that euryhaline invertebrates regulate the concentrations of free amino acids to adjust intracellular osmotic pressure [33].

Besides genes participating in ion and amino acid channels, genes related to immune responses were also found. Stress-induced immune changes have been elucidated in many marine invertebrates [34], including *C. gigas* [35], [36] and mussel [37], [38]. For the invertebrate metazoan, innate immunity is the only immunological defense mechanism [39]. In addition, phagocytosis by immune cells is the predominant mechanism of marine invertebrates defense [34], which is the same to *C. gigas*. In our results, C-type lectin, thioester-containing protein and C1q domain containing proteins were all up-regulated, demonstrating their involvement in innate immunity. Lectins are a family of carbohydrate-recognition proteins that play crucial self- and non-self-recognition roles in innate immunity and can be found in soluble or membrane-associated forms [40]. The C1q domain-containing proteins include a wide range of signaling molecules and are known to participate in the control of inflammation, innate immunity, and energy homeostasis [12]. Down-regulated genes in response to hypoosmotic shock are molecules related to antimicrobial activity, like 2′–5′ oligoadenylate synthetase 1, which is interferon-induced protein and its function is to bind and active a latent endoribonuclease responsible for the degradation of viral and cellular RNAs to impair viral replication [41]. Zinc proteinase regulates many different biological processes, including connective tissue remodeling and removal of signal sequences in nascent proteins [42]. In *Litopenaeus vannamei*, mRNA of zinc proteinase is more expression in WSSV resistant individuals, which proved it played a role in immune responses [43], [44].,. These data may indicate that low salinity shock only triggers the first step of innate immunity that acts by detecting initiating effectors responses, namely pathogen recognition, whereas the antimicrobial defenses are suppressed Meanwhile, high representation of sequences for stress proteins, heat shock protein 70 (HSP70), was also evidenced up-regulated. HSPs, as molecular chaperones, are closely linked to the innate immune system. HSP70 can interfere with the process of apoptotic cell death [45], [46], and it is verified to be up-regulated by stressful conditions in oysters [47].

Our study highlighted Ca^2+^-binding proteins that were all down-regulated significantly such as protocadherin, calmodulin, copine family proteins and calcium-binding EGF domain protein. These calcium-binding genes are involved in the maintenance of calcium homeostasis [48]. In addition, their expressions are relevant to the intracellular Ca^2+^ levels and they take part in the calcium-binding signal transduction [49], [50]. The intracellular Ca^2+^ levels have been taken into account as immune-related parameters [38], [51]. In the immune cell activation, the early key feature is an increase of the intracellular Ca^2+^, which acts as a second messenger of signal transduction [51]. In addition, the intracellular Ca^2+^ concentration is higher with exposure to live bacteria [51]. Calmodulin (CaM) is a calcium-binding protein that modulates many kinds of biological processes affected by cytosolic calcium ions, such as muscle contraction, fertilization, cell proliferation, apoptosis and so on [52]. Moreover, the CaM-dependent signal transduction have been found to play an important role in shrimp defense against pathogen infection in Pacific white shrimp [53]. Furthermore, it has been shown that the concentration of Ca^2+^ dropped with the decrease in Ca^2+^ concentration in the external environment in *C. gigas* [28]. Owing to the lower concentration of Ca^2+^ and no artificial live bacteria stimulus, Ca^2+^-binding signaling pathways and proteins tended to be down-regulated.

Apoptosis, programmed cell death, also called cell suicide, was also evidenced in our data with significant difference. Among them, Baculoviral IAP repeat-containing protein, an apoptosis suppressor, inhibits the caspase activity directly [54]. In addition, IAPs are involved in signal transduction and cell cycle regulation [55]. Those down-regulated genes like caspases are apoptosis triggers or to be active in response to apoptosis triggers. As PD-L1, it acts on nonhematopoietic cells to protect tissues from autoimmune attack [56]. PDCD1, a surface receptor binds to PD-L1, inhibits T-cell proliferation and controls apoptosis process [56]. Here, the expressions of genes related to apoptosis all focused on inhibiting apoptosis. From our data, we can conclude that the ability of apoptosis suppression, namely enhanced cellular survival, may be one of reasons to be euryhalineorganism for *C. gigas*.

The data presented here reveal that genes involved in cell adhesion and communication, signal receptors, and cytoskeleton may play an important role in salinity shock. Cell adhesion is intimately related to the processes of cell motility and cell migration [57]. These genes may contribute to oyster recovery to adjust to the hypoosmotic shock and osmotic stress signaling. Among them, Fer and Fps that expressed significant changes in our data are the only two known members of a distinct subgroup of the protein-tyrosine kinase. Fps may involve a signaling role downstream from multiple cytokine receptors [58]. Fer both plays roles in signaling and involves in regulation of cell-cell interactions and cell-cell adhesion [59], [60]. They may participate in the signal transduction in response to low salinity. LRIG3, as a cell surface receptor, mediates signaling across the plasma membrane [61] and is also found to be the positive regulation of BMP signaling that exquisitely controls homeostatic events [62], suggesting that LRIG3 in our study controls homeostatic events to accommodate the internal osmolality. Collagen is an abundant protein that is part of the extracellular matrix and deposited or broken down as part of the process of tissue growth and repair. Therefore, the reduction of collagen production may imply that more energy would be available to fuel stress responses [63]. In the contrary, the up-regulated gene, ankyrins as a family of ubiquitously expressed membrane adaptor molecules associates with the spectrin-based cytoskeleton network and various membrane proteins [64]. They highly expressed in transcriptome that may be relevant to targeting ion transport proteins and cytoskeleton rearrangement with tubulin and actin (up-regulated genes) to maintain internal and external osmotic pressure balance. In addition, genes involved in cell cycle, like MEGF10 and Protein fosB, also showed changes here. MEGF10 was down-regulated for inhibiting cell motility and cell proliferation in vitro [65]. Protein fosB interacts with c-jun proteins enhancing their DNA binding activity in cellular proliferation [66]. The cell cycle control was suggested to be a key element of the osmotic stress response in blue mussels [27]. Thus, genes involved in the cell cycle may arrest in response to salinity stress in *C. gigas*.

## Conclusions

Using the next-generation sequencing technologies, the present study demonstrated the value of whole transcriptome analysis generated by RNAseq for accurate quantification of gene expression. Through more than 1.5 million sequence reads obtained, we established transcriptome data set for oysters subjected to hypoosmotic stress for the first time, and the data we generated could enrich on genomic resources of this non-model organism. Moreover, we provided a catalog of genes and cellular functions that are potential targets for mechanisms of adaption to low salinity by quantifying gene expression. The results provide not only a better understanding of the molecular mechanisms that control or contribute to the responses to osmotic stress of the oysters, but also an initial look at the cascade of gene expression patterns of marine organisms that occur in response to osmotic stress.

## Materials and Methods

### Oyster materials

Adult individuals of *C. gigas* were purchased from an oyster farm in Weihai, Shandong Province, China, in 2010, and acclimatized over a week in 25‰ filtered seawater at 20°C. The Pacific oyster is not the endangered or protected species, and no specific permits were required for our studies. Twenty-four oysters were individually tagged and divided into two groups in separate tanks. One group was exposed in the optimal salinity filtered seawater (25‰), as the control group (PC); another group was exposed in the simulated conditions of increased fresh water input (8‰), as the treated group (PT). A part of the shell edge (about 10 mm long and 5 mm wide) of each specimen was chipped away in order to ensure the free exchange of seawater between the inside and outside of the shell. Each group was distributed in two tanks, and disposed for 8 hours simultaneously. After that, six oysters of each group were chosen randomly to be sacrificed, and their gill tissues were dissected and saved in RNA store (Dongsheng Biotech.).

### RNA extraction

The gills from 6 specimens saved in RNA store of each group were balanced mixed. Each sample was lysed in 1 ml of TRIzol reagent (Invitrogen) for total RNA extraction according to the manufacturer's instructions. Total RNA concentration was measured on a NanoDrop (Thermo Fisher Scientific) and RNA integrity [67] was checked on an Agilent 2100 BioAnalyzer (Agilent Technologies). Total RNA was treated by Dnase I (Ambion) following the manufacturer's protocol, and Poly-A RNA was extracted from each total RNA sample using MicroPoly(A)Purist™ Kit (Ambion) according to manufacturer's instructions. Equal quantities of high-quality mRNA from each material were pooled for cDNA synthesis.

### cDNA library construction and sequencing

cDNA samples were prepared following the protocol described in [68]. Briefly, first-strand cDNAs were synthesized using SuperScript II reverse transcriptase (Invitrogen) with Gsul-oligo-dT primer. The second-strand cDNAs were synthesized using Ex Taq polymerase (Takara). After the double-stranded cDNAs were synthesized, the cDNAs were cleaved off by Gsul restriction enzyme.

The synthetic cDNA was mode to 300–500 bp by a UTR200 sonication device (Hielscher Ultrasonics GmbH), and was selected using AMpure beads (Agencourt). This was followed by amplifying with TruSeq PE cluster kit v3-cBot-HS (Illumina) and constructing libraries with TruSeq™ DNA sample prep kit-set A (Illumina) according to the manufacturer's instructions. In the end, the cDNA library was sequenced on the Illumina sequencing platform (HiSeq 2000) from both ends of each fragment.

### Sequence data analysis and functional annotation

Raw data generated from Illumina sequencing were preprocessed to remove nonsense sequences and were assembled as contigs by Velvet [69] and Oases [70] software. The assembled sequences were predicted to mapping to protein-coding sequences by GetORF of EMBOSS [71]. The predicted protein-coding sequences searched against the Swiss-Prot database, the NCBI non-redundant (Nr) protein database and the database of sea hare using Blastp with an E-value of 1e-5. Gene names were assigned to each protein sequences based on the best BLAST hit.

To annotate the assembled sequences with GO terms, which have three main categories (cellular component, biological process and molecular function), the Swiss-Prot and its supplement TrEMBL BLAST results were imported into GoPipe [72] software according to gene2go to retrieve GO terms. These GO terms were assigned to query protein sequences, producing a broad overview of groups of genes cataloged in the three ontology vocabularies. What's more, the data presented herein represented a GO analysis at level 2, illustrating general functional categories. KEGG pathways were assigned to the assembled sequences using the online KEGG Automatic Annotation Sever (Kyoto encyclopedia of genes and genomes, http://www.genome.jp/kegg/kaas/) using bi-directional best-hit method [73]. This server provides KEGG Orthology (KO) assignments and pathway mapping.

### Gene expression difference analysis

Firstly, we counted the number of reads from two samples that can be mapped to the assembly contigs respectively and transformed into RPKM (Reads Per Kilo bases per Million reads) [74]. Then we calculated express abundance differences of each gene that was on behalf of a contig between two samples using MARS (MA-plot-based method with Random Sampling model) in the package of DEGseq [75] with FDR <0.001 and the absolute value of fold change >2.

## Supporting Information

Table S1 **List of functional groups and related gill up-regulated genes from oysters in response to hypoosmotic stress** ***versus*** **in optimal salinity conditions.**
(XLSX)

Table S2 **List of functional groups and related gill down-regulated genes from oysters in response to hypoosmotic stress** ***versus*** **in optimal salinity conditions.**
(XLSX)
